# Gas phase dehydration of glycerol to acrolein over WO_3_-based catalysts prepared by non-hydrolytic sol–gel synthesis†

**DOI:** 10.1039/c8ra01575a

**Published:** 2018-04-10

**Authors:** L. Nadji, A. Massó, D. Delgado, R. Issaadi, E. Rodriguez-Aguado, E. Rodriguez-Castellón, J. M. López Nieto

**Affiliations:** Instituto de Tecnología Química, Universitat Politècnica de València-Consejo Superior de Investigaciones Científicas Avenida de los Naranjos s/n 46022 Valencia Spain jmlopez@itq.upv.es; Laboratoire des Applications Energétiques de l'Hydrogène, Faculté de Technologie, Université Saad Dahlab Blida 1 Algeria; Department of Inorganic Chemistry, University of Malaga Campus de Teatinos 29071 Málaga Spain

## Abstract

Solid acid catalysts based on WO_3_–SiO_2_ and WO_3_–ZrO_2_–SiO_2_ were prepared by one-pot non-hydrolytic sol–gel method and tested in the gas phase glycerol dehydration to acrolein. Their structural and textural characteristics were determined by X-ray diffraction (XRD), N_2_ adsorption, X-ray energy dispersive spectroscopy (XEDS), Fourier transform infrared spectroscopy (FTIR), Raman spectroscopy and X-ray photoelectron spectroscopy (XPS). Their acid characteristics were studied by both temperature programmed desorption of ammonia (NH_3_-TPD) and FTIR of adsorbed pyridine. Under our operating conditions, all the catalysts were active and selective in the transformation of glycerol to acrolein, which was always the main reaction product. The high selectivity to acrolein is achieved on catalysts presenting a higher proportion of Brønsted acid sites. In addition, the role of oxygen in the feed on catalytic performance of these catalysts is also discussed.

## Abstract 2

Active and selective W–Si–(Zr)–O catalysts for glycerol dehydration to acrolein have been successfully prepared by non-hydrolytic sol gel method.

## Graphical abstract

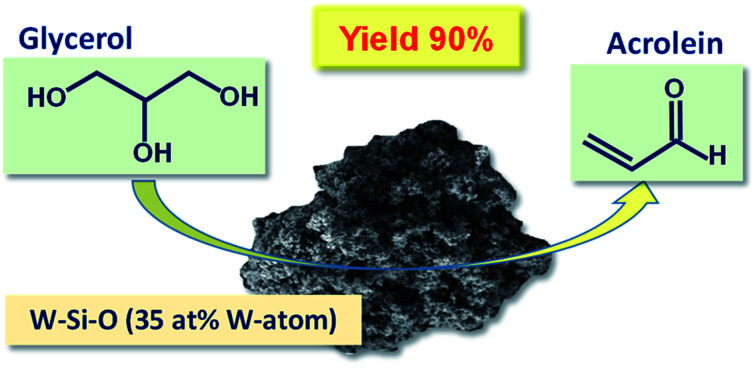

## 1. Introduction

The utilization of biomass as renewable feedstock for the substitution of fossil sources constitutes a promising alternative in moving our economy toward a more sustainable future.^1–3^ Nowadays, about 85% of world commercial energy is obtained from fossil fuels and biodiesel.^2^ During biodiesel production glycerol is the main by-product obtained, representing *ca.* 10 wt%. Therefore, the use of glycerol for the production of added value chemicals could partially compensate the higher production costs of biodiesel compared to petrodiesel.^4–7^ A potential use of glycerol is the obtention of acrolein which is an important intermediate in the chemical industry for the production of acrylic acid, esters, methionine, fragrances, polymers or detergents.^4–7^

The current industrial process to obtain acrolein is the gas phase oxidation of propylene with a Bi/Mo-mixed oxide catalyst, in which the selectivity to acrolein can reach 85% at a propylene conversion of 95%.^8^ Moreover, an alternative route could be the gas phase transformation of glycerol to acrolein over solid acid catalysts by a dehydration mechanism.^4–7,9–19^ In this case, Brønsted acids sites are more active and selective than Lewis acid sites,^13^ and the reaction takes place *via* a two-step process that starts with the intramolecular dehydration of the internal hydroxyl group of glycerol, followed by tautomerization and subsequent dehydration of the terminal hydroxyl group.^4–7,9–19^

Several type of catalysts have been proposed in the last years,^4–7^ including zeolites,^9,10^ heteropoly acids and acidic heteropoly salt,^11^ phosphate-based materials^12^ and oxides.^13–21^ Among these, W-containing materials (both bulk and supported ones),^15–21^ are promising catalysts due to their high selectivity to acrolein and catalyst stability. Nevertheless, the deactivation of the catalyst during the reaction is one of the major drawbacks.^4–7,9–21^ In this regard, Dubois *et al.*^17^ suggested the *in situ* regeneration of the catalyst by co-feeding molecular oxygen. In addition, it has been also observed that oxygen not only decreases the coking rate, but also improves the selectivity to acrolein.^17,18^ The beneficial effect of oxygen, reducing the formation of coke precursors and heavy compounds, has already been observed in several catalytic systems.^17,18,18*a*^

Among all the catalysts proposed for glycerol dehydration to acrolein, supported tungsten oxide catalysts are one of the most efficient.^14–21^ Although they are usually prepared by impregnation, the sol–gel method also appears as an attractive preparation route since it allows the synthesis of mixed oxide catalysts in one-step. Maksasithorn *et al.*^22^ reported the preparation of W–Si and W–Si–Al metathesis catalyst by sol–gel method which proved to be more active and selective than a catalyst of similar composition obtained by impregnation.

Non-hydrolytic sol–gel (NHSG) route based on the reaction of chloride or alkoxide precursors with diisopropyl ether has shown to provide an excellent control over the stoichiometry and homogeneity of mixed oxide gels.^22–26^ This ether route is based on the *in situ* formation of alkoxide groups by reaction of the halide groups with the ether, followed by non-hydrolytic condensation between these alkoxide groups and the remaining chloride groups.^26^ Owing to the generally high degree of condensation of non-hydrolytic gels, mesoporous xerogels with high surface area and pore volume can be obtained by simple evaporative drying in the absence of templating agent. Thus, these non-hydrolytic routes are attracting increasing attention for the preparation of W-containing mixed oxide catalysts.^22–25^

In the present paper, we describe the one-step NHSG preparation of WO_3_–SiO_2_ and WO_3_–ZrO_2_–SiO_2_ materials, in which the W-content is varied in a systematic way. We pay special attention toward effect of zirconium addition in the ternary WO_3_–ZrO_2_–SiO_2_ formulation oxide. These catalysts were characterized by N_2_-physisorption, XRD, NH_3_-TPD, FTIR of adsorbed pyridine, XEDS, and FTIR, Raman and XPS spectroscopies and tested in the dehydration of glycerol to acrolein.

## 2. Experimental

### 2.1. Catalysts preparation

WO_3_–SiO_2_ and WO_3_–ZrO_2_–SiO_2_ catalysts have been prepared by the non-hydrolytic sol–gel method, under argon atmosphere inside a glove box according to a method previously reported,^22–26^ using SiCl_4_ (Sigma Aldrich, 99.9%), ZrCl_4_ (Sigma Aldrich, 99.9%), WCl_6_ (Sigma Aldrich, 99.5%), anhydrous dichloromethane (CH_2_Cl_2_, Aldrich, 99.8%) and diisopropyl ether (iPr_2_O, Aldrich, 99%) as reactants. The synthesis was carried out in Teflon-lined stainless steel autoclaves of 300 ml by reacting chloride precursors (WCl_6_, SiCl_4_ and/or ZrCl_4_) with diisopropyl ether, using an iPr_2_O/metal chloride molar ratio of 1. Then, the solvent (20 ml of dichloromethane) was introduced. Finally, the mixture was heated-treated at 110 °C for 1 day under autogenous pressure (*ca.* 0.6 MPa). After 24 hours, the autoclave was cooled down to room temperature; and the gel was washed three times with dichloromethane and dried at 20 °C under nitrogen atmosphere overnight. The xerogel was finally crushed in a mortar and calcined in a muffle for 3 h at 500 °C (heating rate 3 °C min^−1^). WO_3_–SiO_2_ and WO_3_–ZrO_2_–SiO_2_ samples are named as *x*W–Si and *x*W–*y*ZrSi, respectively, where *x* represents the nominal W-content (in % of W atom), and *y* represents the Zr/(Zr + Si) atomic ratio. For comparison, WO_3_–ZrO_2_ samples will be named as *x*W–Zr.

### 2.2. Characterization of catalysts

Powder X-ray diffraction (XRD) was used to identify the crystalline phases present in the catalysts. XRD patterns were obtained in a PANalytical X'Pert PRO diffractometer with a X'Celerator detector in Bragg–Brentano geometry using CuKα radiation.

X-Ray energy dispersive spectroscopy (XEDS) was carried out in a Zeiss Ultra-55 field emission scanning electron microscope, which was equipped with an Oxford LINK ISIS X-Ray detector. Spectra were collected at an accelerating voltage of 12 kV at a counting time of 100 s.

N_2_-adsorption isotherms were collected in a Micromeritics ASAP 2020 instrument. Samples were degassed at 400 °C prior to N_2_ adsorption. Surface areas and pore volumes were calculated by Brunauer–Emmet–Teller (BET) and Barret–Joyner–Halenda (BJH) methods, respectively.

The total acidity of fresh catalysts was evaluated by means of temperature-programmed desorption of ammonia (TPD-NH_3_). The TPD-NH_3_ profiles were obtained by placing 80 mg of catalyst into a tubular reactor. The adsorption of ammonia (5 min) at 100 °C was performed after cleaning with helium (35 ml min^−1^) from room temperature to 550 °C, and cooling to 100 °C. After adsorption, helium flow (35 ml min^−1^) was passed to eliminate the physisorbed ammonia. Finally, thermo-programmed desorption was carried out by heating the samples from 100 to 550 °C with a heating rate of 10 °C min^−1^. The evolved ammonia was analysed by an on-line gas chromatograph (Shimadzu GC-14A) provided with a TCD. In order to quantify the amount of ammonia desorbed, the equipment was previously calibrated.

Fourier Transform Infrared Spectroscopy (FTIR) was performed in a Nicolet 205xB spectrometer. Typically, 20 mg of dried sample were mixed with 100 mg of potassium bromide (KBr) and pressed to obtain a pellet. Spectra were collected in the 300–4000 cm^−1^ region at a spectral resolution of 1 cm^−1^ and 128 accumulations per scans.

FTIR of adsorbed pyridine was performed in a Nicolet 710 FT IR spectrometer. The catalysts were pressed into self-supported wafers of 10 mg cm^−1^ and activated at 400 °C in vacuum for 1 h. Then pyridine was introduced into the cell and, when equilibrium was reached, catalysts were outgassed at 150 °C and cooled to room temperature. Then the spectra were recorded. The concentration of Brønsted and Lewis sites was calculated from integrated intensities and the corresponding extinction coefficients using the method proposed by Emeis.^27^

Raman spectra were collected in an inVia Renishaw spectrophotometer, equipped with an Olympus microscope and a Renishaw HPNIR laser. Samples were excited at 514 nm (corresponding to visible green light in the electromagnetic spectrum) with a power of 15 mW on the samples.

X-Ray photoelectron spectroscopy (XPS) was performed on a SPECS spectrometer with a Phoibos 150 MCD-9 detector, using a non-monochromatic Al Kα X-ray source (1486.6 eV). Spectra were recorded at 50 eV of pass energy, an X-ray power of 200 W and an operating pressure of 10^−9^ mbar. Binding energy values were referenced to C 1s peak (284.5 eV). Data treatment was performed with CasaXPS software.

### 2.3. Catalytic tests

The gas phase transformation of glycerol was carried out at atmospheric pressure in a fixed-bed reactor at a contact time, W/F, of 110 g_cat_ h (mol_GLY_)^−1^, in the range of 280–380 °C. The feed consisted of a mixture glycerol/water/oxygen/helium with a molar ratio of 2/40/4/54 (aerobic conditions) or 2/40/0/56 (anaerobic conditions). The effluent stream was bubbled through a condenser device at 0–3 °C, while the remaining gaseous products were analysed by online gas chromatography (HP 6890) equipped with two chromatographic columns:^28^ (i) a molecular sieve 5 Å (3 m length); and (ii) a Porapak Q (3 m) column. The condensed liquids were analysed by gas chromatography in a Varian 3900 chromatograph equipped with a 100% dimethylpolysiloxane capillary column (100 m × 0.25 mm id; 0.5 μm).

## 3. Results and discussion

### 3.1. Catalyst characterization

Table 1 shows the main characteristics of W-containing catalysts. For comparison, the characteristics of W-free SiO_2_–ZrO_2_ samples are shown in Table S1.† In general, the experimental molar composition of the materials, determined by XEDS, is in agreement with the theoretical synthesis formulation, confirming that NHSG provides a good control over the stoichiometry of the mixed metal oxides (Table 1).

**Table 1:** Physicochemical characteristics of catalysts prepared by NHSG

Catalyst	Composition (XEDS)		Surface area, m^2^ g^−1^	Pore diameter, nm	TPD-NH_3_, μmol_NH_3__ g^−1^	B/(B + L), FTIR (pyridine)	Conversion^a^ (%)	Selectivity^a^ (%)
	W (at%)	Zr/(Zr + Si)						
0W–Si	0	0	583	11.9	107	n.d.	71.0	8.3
5W–Si	2.5	0	773	2.6	455	0.19	90.8	76.8
15W–Si	13.8	0	474	5.7	564	0.21	100	82.2
35W–Si	31.9	0	276	5.3	371	0.29	100	90.1
50WSi	55.5	0	140	6.6	n.d.	0.15	100	74.0
75W–Si	72.3	0	67	11.4	n.d.	n.d.	100	74.4
WO_3_	100	0	30	7.2	57.2	n.d.	99.8	79.0
0W–10ZrSi	0	10.1	334	42	860	n.d.	78.2	67.8
5W–10ZrSi	4.6	9.1	635	5.4	825	n.d.	88.4	76.1
15W–10ZrSi	10.6	9.5	438	9.2	817	0.18	99.9	79.1
35W–10ZrSi	36.6	12.2	267	7.3	790	0.40	99.1	83.8
50W–10ZrSi	45.8	12.2	113	8.2	604	n.d.	99.6	76.5
75W–10ZrSi	71.2	10.4	79	9.1	n.d.	n.d.	100	76.9
15W–20ZrSi	13.9	18.0	334	7.1	712	n.d.	100	75.0
15W–40ZrSi	15.1	39.8	231	6.8	590	0.31	100	82.3
15W–Zr	13.3	100	141	7.8	378	n.d.	100	70.2

a Data measured after 90 min on stream. Experimental conditions: 0.3 g catalyst; contact time, W/F, of 110 g_cat_ h (mol_GLY_)^−1^; glycerol/H_2_O/O_2_/He molar ratio of 2/40/4/54.

N_2_-adsorption isotherms of calcined catalysts are shown in Fig. S1 (ESI†), whereas the main textural properties of the catalysts are summarized in Table 1. Adsorption–desorption profiles show hysteresis loops typical of mesoporous materials,^29^ giving average pore sizes in the 3–11 nm range (Table 1). The specific surface area and pore volumes of catalysts strongly depend on the W and/or Zr content. In this way, both the surface area and the pore volume initially increase when low tungsten amount is added (5W–Si and 5W–10ZrSi), but they decrease dramatically with the W-loading for samples with high W-contents. The same trend is observed for both *x*W–Si-and *x*W–10ZrSi series. In addition, when comparing samples with different Zr-content (Table 1 and S1†), it can be concluded that the surface area of catalysts decreases also when increasing the Zr-loading.

**Fig. 1:**
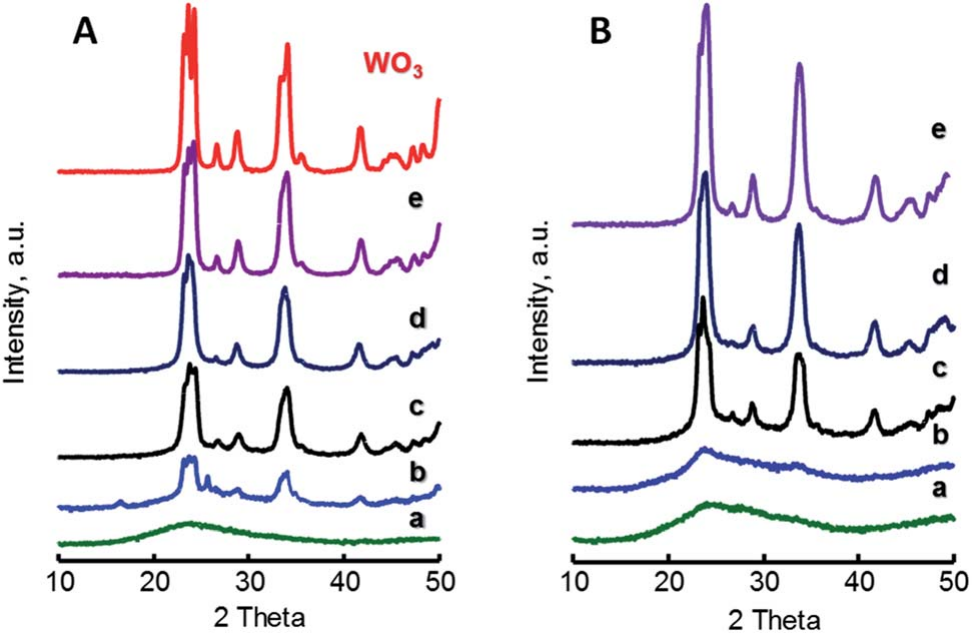
XRD patterns of *x*W–Si (A) and *x*W–10ZrSi (B) catalysts with different W-contents: 5% (a); 15% (b); 35% (c); 50% (d); and 75 at% of W-atoms. For comparison it has been also included the XRD pattern of pure WO_3_. Characteristics of catalyst in Table 1.

Fig. 1 shows the X-ray diffraction patterns of W-containing catalysts for both *x*W–Si (Fig. 1A) and *x*W–10ZrSi (Fig. 1B) series. For comparison, XRD patterns of other W–Zr–Si–O and W–Zr–O catalysts as well as W-free Zr–Si–O materials are presented in Fig. S2 (ESI†). In the case of *x*W–Si series (Fig. 1A), monoclinic WO_3_ phase are observed (*m*-WO_3_, main peaks at 2*θ* = 23.1, 23.6, 24.4 and 34.2°, space group: *P*2_1_/*n*).^30^ The relative presence of this phase increases with W-loading in the samples, being absent in the material with the lowest W-content, *i.e.* sample 5W–Si (Fig. 1A, pattern a). The absence of tungsten oxide reflections can be explained by the low W content and the high dispersion of tungsten species in this catalyst.

A similar trend is observed for *x*W–10ZrSi series (Fig. 1b), in which *m*-WO_3_ is the only crystalline phase observed, although tungsten oxide reflections have not been found in the sample with W-loading of 5 at% but neither in that of 10 at% (Fig. 1B, patterns a and b). This suggests that the Zr–Si–O can disperse tungsten oxide better than silica in spite of its lower surface area. This better dispersion can be corroborated by comparing the XRD patterns of samples with 15 at% of W but different Zr/Si ratio, *i.e.* 15W–*y*ZrSi catalysts (Fig. S2-A†) since the crystallinity of *m*-WO_3_ phase decreases when increasing the Zr-content, suggesting a possible interaction between W and Zr atoms, thus facilitating the dispersion of W^6+^ species (Fig. S2†).

This interaction could prevent the formation of well-ordered tungsten or zirconium oxide phases, as it was previously reported for other systems based on W and Zr oxides.^31^ In fact, well-crystallized phases are only obtained in Zr-free materials, *i.e. m*-WO_3_ in sample 15W–Si (Fig. 1A, pattern b), or in Si-free materials, *i.e.* tetragonal *t*-ZrO_2_ (JCPDS: 80–0965) in sample 15W–Zr (Fig. S2-B†). In addition, XRD analyses of W-free Zr–Si–O samples (0W–*y*ZrSi series) show that, in the absence of W, the crystallization of *t*-ZrO_2_ and monoclinic *m*-ZrO_2_ (JCPDS: 37–1484) is favoured when increasing the Zr-content (Fig. S2-C†).^32^

**Fig. 2:**
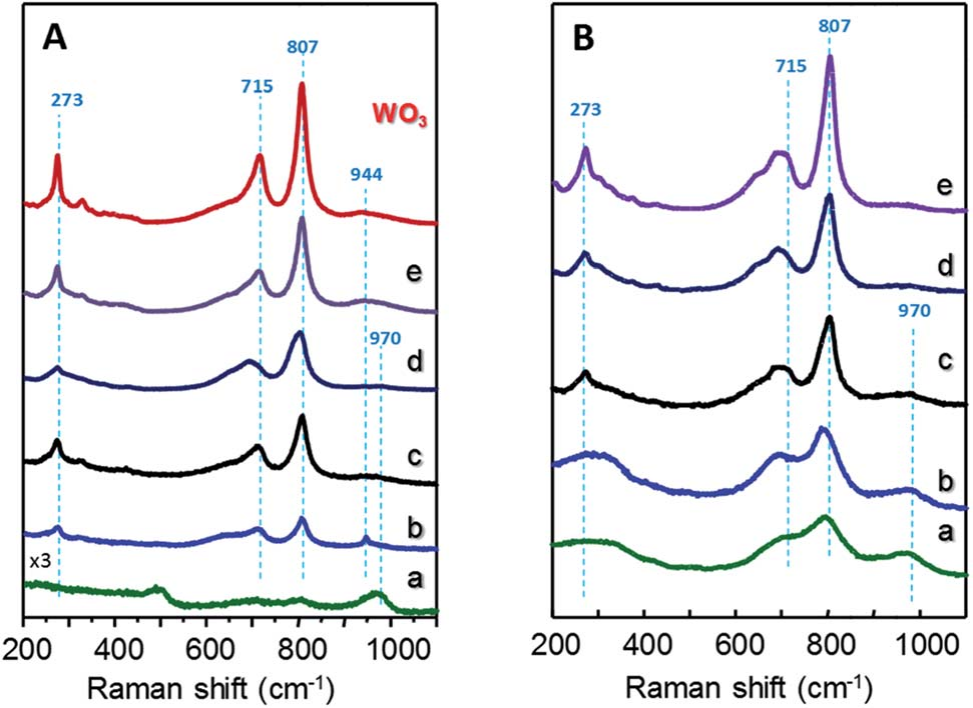
Raman profiles of *x*W–Si (A) and *x*W–10ZrSi (B) catalysts with different W-contents: 5% (a); 15% (b); 35% (c); 50% (d); and 75 at% of W-atoms. For comparison, it has been also included the Raman spectrum of pure WO_3_.

Fig. 2 shows Raman spectra of *x*W–Si (Fig. 2A) and *x*W–10ZrSi (Fig. 2B) catalysts. For comparison, the spectra of 15W–*y*ZrSi series (*y* = 20, 40 or 100) are also shown in Fig. S3.† Raman bands at 970, 807, 715 and 273 cm^−1^ are observed in both *x*W–Si (Fig. 2A) and *x*W–10ZrSi (Fig. 2B) catalysts, which can be attributed to the presence of WO_3_ particles.^17,33,34^ Raman bands at 807 and 715 cm^−1^ are assigned to W–O–W stretching modes, while the band at 273 cm^−1^ can be assigned to W–O–W bending modes in the tungsten oxide framework. Another band appearing at 970 cm^−1^ is also observed in all the catalysts, which has been assigned to W=O stretching modes of terminal W=O bonds.^17,35–38^ It is worth mentioning that increasing W-content in both *x*W–Si and *x*W–10ZrSi series gives rise to an increase in the relative intensity of the signals corresponding to W–O–W vibrational modes (*i.e.* bands at 807, 715 and 273 cm^−1^) with respect to the band at 970 cm^−1^ (ascribed to W=O bonds). This means that the proportion of terminal W=O species in the catalyst is higher at lower W content, which is consistent with a higher dispersion of the tungsten oxide formed (but also a lower crystallite size). In this way, higher surface areas and higher dispersion of WO_3_ are achieved at lower W-loading (Table 1). In the case of sample with similar W-content and different Zr/Si ratio, *i.e.* 15W–*y*ZrSi series, similar Raman bands due to the presence of WO_3_ particles are observed at relatively low Zr contents (Fig. S3†). Interestingly, these bands show a broader profile, which is in agreement with the amorphous nature of the catalysts observed by XRD. On the other hand, the Raman profile is completely different for the Si-free catalyst, sample 15W–ZrO_2_ (Fig. S3,† pattern d): (i) no bands related to *m*-WO_3_ are observed; (ii) it presents signals at 310, 475, 606 and 643 cm^−1^ (Fig. 2c), related to tetragonal *t*-ZrO_2_ phase;^39^ and (iii) two extra bands at 944 and 840 cm^−1^, which can be attributed to symmetric and asymmetric W=O stretching modes, respectively, are observed.^35^ All these results suggest a relatively high interaction between W and Zr atoms, preventing the formation of tungsten oxide crystalline phases at low W-loadings.^35^

Important differences have been also observed in the FTIR spectra of these catalysts (Fig. S4†). All the samples show two bands around 3450 and 1630 cm^−1^, which are assigned to stretching and bending O–H modes respectively,^40–42^ although the band at 1630 cm^−1^ can also be due to the presence of physisorbed water. On the other hand, bands at 815 and 948 cm^−1^ in W-containing materials are assigned to W–O–W bridge-type and W=O stretching-type vibrations, respectively (Fig. S4-A and B†).^24^ Si-containing catalysts show additional bands at *ca.* 1180, 1060 and 450 cm^−1^ that can be ascribed to Si–O bond vibrations (Fig. S4†),^41^ whose relative intensity increases with the amount of Si in the materials. In the case of Zr-containing samples, signals related to zirconium oxide are only found in the 15W–Zr sample, being absent in the other catalysts.

The acid properties of W-containing catalysts have been studied by means of TPD-NH_3_ and FTIR spectroscopy of adsorbed pyridine. Measuring the amount of adsorbed ammonia allows us to determine the total concentration of surface acid sites. Also, by analysing the desorption profiles measured at increasing temperatures it is possible to estimate the specific acid strength of those sites. Thus, NH_3_ will be desorbed at higher temperatures as the strength of the acid sites increases.

Considering the total concentration of acid sites in *x*W–Si series, measured by TPD-NH_3_, it is noteworthy to mention that a maximum number of acid sites is found for 15W–Si catalyst (564 μmol_NH_3__ g^−1^) (Table 1). Then, the amount of acid sites progressively decreases for catalysts with higher W contents. However, if we consider the surface density of acid sites, *i.e.* the number of acid sites per surface area, we can conclude that it increases when the W-loading increases (Table S2†). These features are in agreement with the decrease in surface area observed at increasing W-loadings and the low acid characteristics of the undoped SiO_2_ sample (Table 1). In the rest of the series, *i.e. x*W–10ZrSi and 15W–*y*ZrSi series, the incorporation of W and/or Zr promotes a decrease in the total concentration of acid sites and an increase in the surface density of acid sites (Table S2†).

**Fig. 3:**
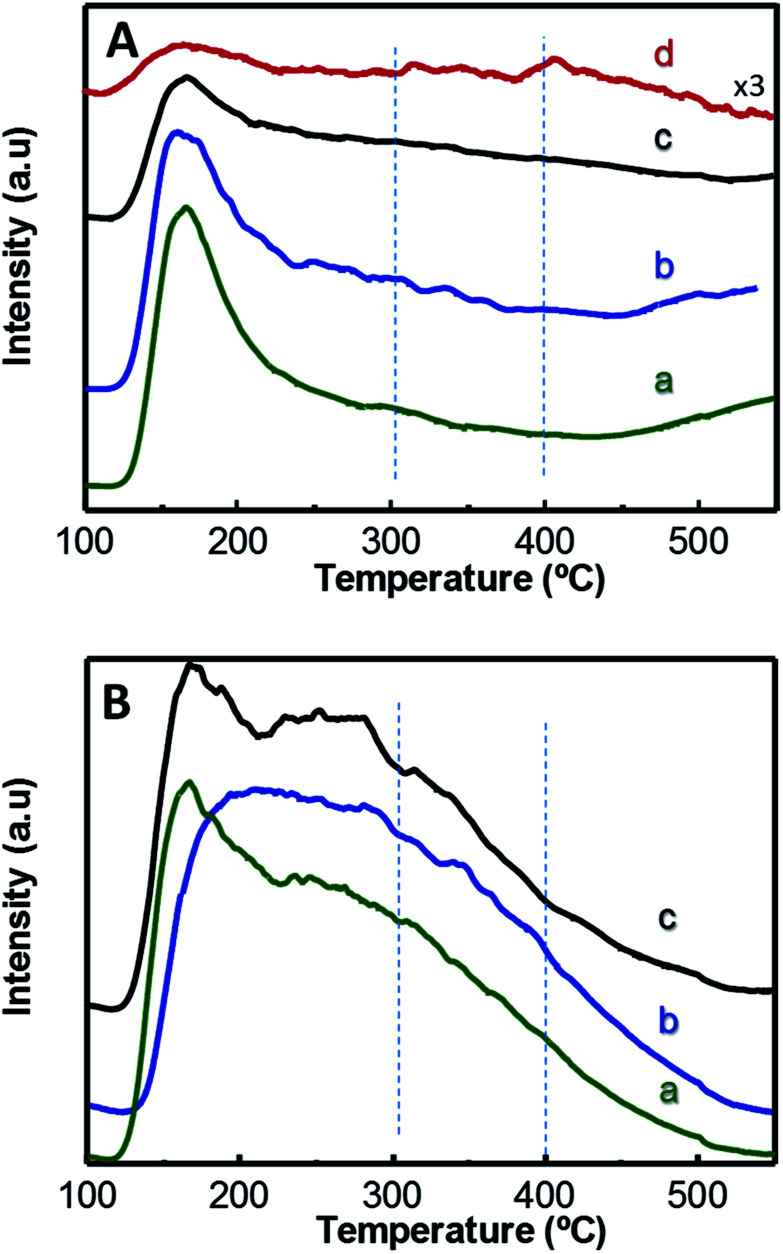
TPD-NH_3_ patterns of *x*W–Si (A) and *x*W–10ZrSi (B) catalysts with different W-contents: 5% (a); 15% (b); 35% at% of W-atoms (c). For comparison it has been also included the TPD-NH_3_ pattern of pure WO_3_ (d).

Fig. 3 displays TPD-NH_3_ profiles of W-containing materials. Catalysts show two main desorption zones centred at approximately 170 °C and 270 °C, which can be attributed to NH_3_ desorbed from acid sites with low and medium–high acid strength, respectively. In this way, Zr-containing catalysts, *i.e. x*W–10ZrSi series (Fig. 3B) and 15W–*y*ZrSi series (Fig. S5†), present a higher contribution to the TCD-signal at higher temperatures (*ca.* 270 °C) than those of *x*W–Si series (Fig. 3A), suggesting that the acid sites with medium–high acid strength mainly correspond to Zr species whereas the low acid strength sites are manly related to the tungsten species. Overall, the introduction of Zr^4+^ ions increases not only the amount of acid sites of these materials but also their acid strength.

On the other hand, FTIR of adsorbed pyridine has been used to study the chemical nature of acid surface sites in the catalysts:^17,43,44^ when pyridine is coordinatively bonded to Lewis sites, the spectrum shows a band at 1440–1460 cm^−1^; whereas when pyridinium ion is formed on the surface, due to its interaction with Brønsted-type acid sites, it shows a band around 1535 cm^−1^. Hence, the concentration of both Brønsted and Lewis acid sites can be calculated from the corresponding band intensities and extinction coefficients of each type of site.^28^

**Fig. 4:**
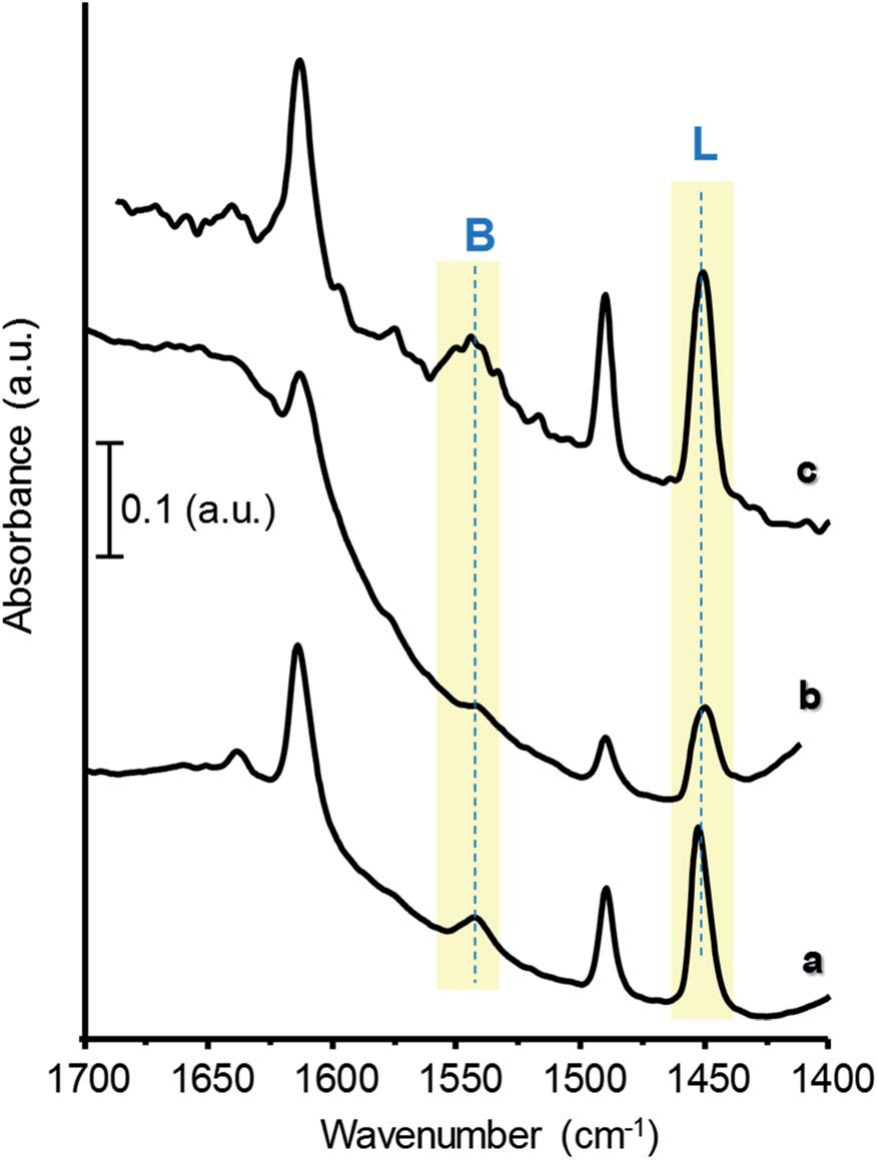
FTIR spectra of adsorbed pyridine of selected catalysts: 15W–Si (a); 50W–Si (b); 35W–10ZrSi (c).

Fig. 4 presents FTIR spectra of adsorbed pyridine of selected catalysts, after evacuation at 150 °C. For comparison, Fig. S6† shows the corresponding spectra of W-free samples. All the materials display bands related to Lewis (L, at *ca.* 1450 cm^−1^) and to Brønsted acid sites (B, at *ca.* 1540 cm^−1^), showing different relative intensities depending on catalysts composition. Samples with high W and/or Zr-loadings show the lowest B/(B + L) acid sites ratio, due to an increase in the concentration of Lewis sites (Table 1, Fig. 4 and S6†). The highest B/(B + L) ratio has been observed for the samples the 35W–Si and 35W–10ZrSi, which, as it will be discussed later, are the most selective catalysts in the dehydration of glycerol.

**Fig. 5:**
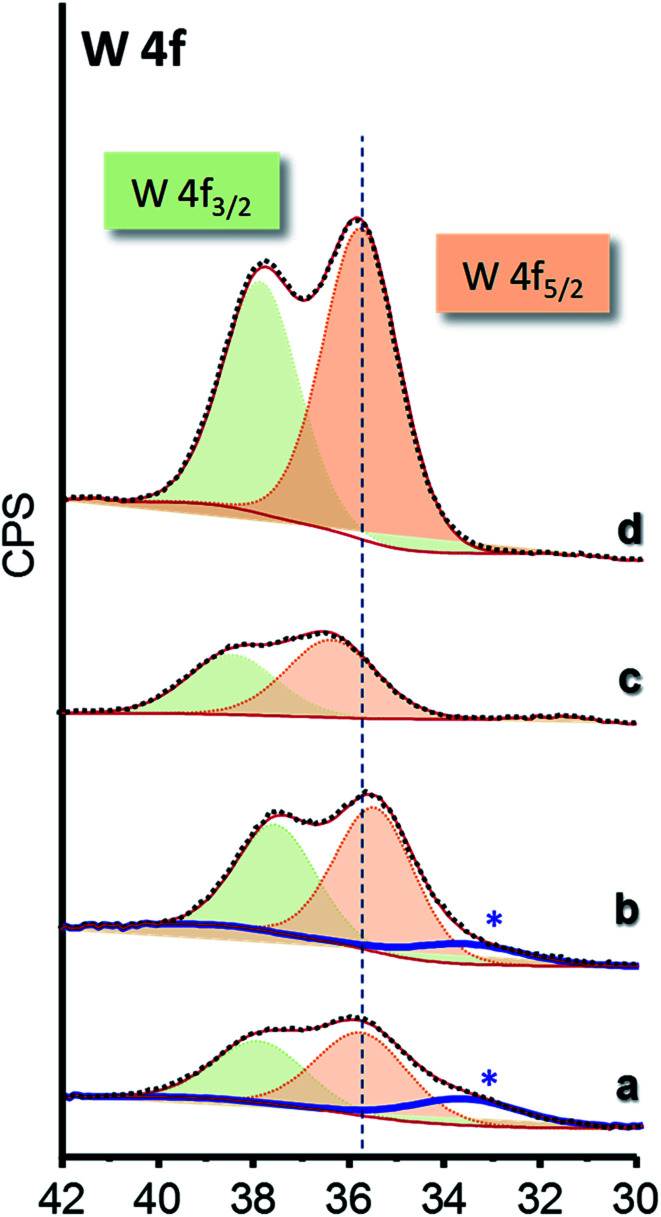
W 4f core-level XPS spectra of selected WO_3_–ZrO_2_–SiO_2_ catalysts: 15W–Si (a); 35W–Si (b); 15W–10ZrSi (c); and 35W–10ZrSi (d).

The chemical nature of surface species of selected catalysts was elucidated by XPS (Fig. 5). W 4f core-level spectra of all the materials show a single W 4f_7/2_ peak at 35.9–36.3 eV which can be assigned to W^6+^ species (Fig. 5 and S7†).^45,46^ Samples of *x*W–Si series present an additional component at lower binding energy, which can be ascribed to differential charging (Fig. 5a and b, marked with an asterisk), due to heterogeneities in the electrical conductivity in the materials. Indeed, this fact has already been reported for W-promoted silica catalysts, in which the lower the W-loading, the higher the differential charging effect.^47^

### 3.2. Catalytic tests

The WO_3_–SiO_2_ and WO_3_–ZrO_2_–SiO_2_ based catalysts prepared by the NHSG method have been tested in the gas phase aerobic transformation of glycerol. The catalytic results obtained at 300 °C on W-containing catalyst, *i.e. x*W–Si, *x*W–10ZrSi and 15W–*y*ZrSi series, are comparatively presented in Tables S3 and S4,† whereas the values of glycerol conversion and selectivity to acrolein are summarized in Table 1. For comparison, binary (WO_3_, SiO_2_ or ZrO_2_) and mixed (Zr–Si–O) oxides have been tested, and their catalytic results are also shown in Table S5.†

On pure SiO_2_ or ZrO_2_, *i.e.* samples 0W–Si and 0W–Zr, respectively, the selectivity to acrolein is very low (8% and 14.5%, respectively) (Table S5†). However, the selectivity to acrolein increases for Zr–Si–O samples with intermediate Zr/Si ratios. Thus, for samples of Zr–Si series, a maximum selectivity to acrolein of 68% was achieved on sample 0W–10ZrSi, whereas it decreases when increasing the Zr-content. The selectivity to acrolein of Zr–Si–O catalysts is higher to those described for Si–Al–O catalysts.^48^

On the other hand, pure tungsten oxide, WO_3_, shows high catalytic activity and relatively high selectivity to acrolein, with a yield to acrolein of *ca.* 79% (Table S5†), which is in agreement to previous results.^28^ In the case of W-containing catalysts, acrolein was the main reaction product under our experimental conditions (Table 1). In addition, acetaldehyde, acetic acid, acrylic acid, CO and CO_2_ were also observed, as minorities (Tables S3 to S5†). Finally, minor amounts of other by-products have been also detected, which have been grouped under the label “others”.

**Fig. 6:**
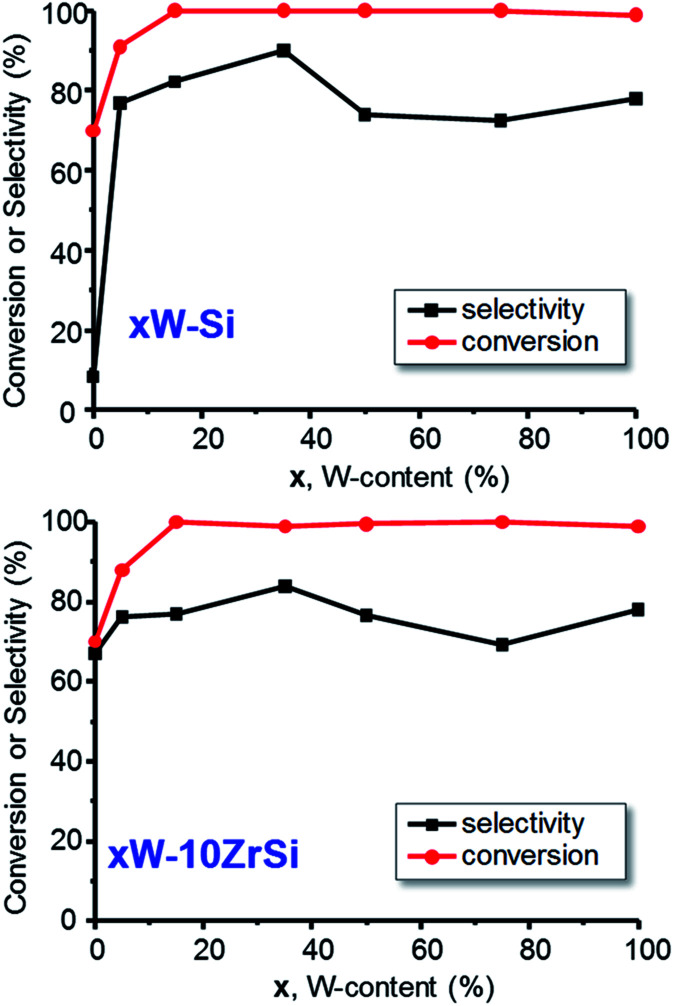
Variation of the glycerol conversion and selectivity to acrolein with the W-content in *x*W–Si (up) and in *x*W–10ZrSi catalysts (down). Experimental conditions: 0.3 g catalyst, contact time, W/F, 110 g_cat_ h (mol_GLY_)^−1^; glycerol/H_2_O/O_2_/He molar ratio of 2/40/4/54.

Fig. 6 shows the variation of the glycerol conversion and the selectivity to acrolein with W-content in the catalysts of *x*W–Si (Fig. 6A) and *x*W–10ZrSi (Fig. 6 B) series. Except in the case of 5W–Si catalysts, a glycerol conversion of 100% is observed under our reaction conditions. The selectivity to acrolein initially increases with W-loading, achieving a maximum selectivity of *ca.* 90% over the sample 35W–Si. In this way, it is worth noting that the incorporation of tungsten limits the formation of heavy compounds and other by-products (see Tables S3 and S4†) in comparison with those observed for W-free Zr–Si–O samples (Table S5†).

**Fig. 7:**
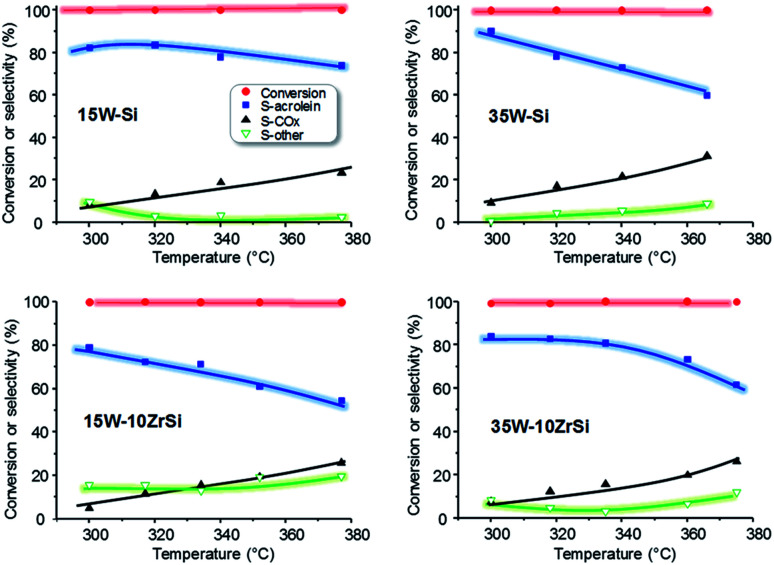
Variation of glycerol conversion and selectivity to acrolein, CO_X_ and other reaction products with time on steam during the aerobic glycerol dehydration over WO_3_–SiO_2_ (*i.e.* samples 15W–Si and 35W–Si) and WO_3_–ZrO_2_–SiO_2_ (*i.e.* samples 15W–10Zr–Si and 35W–10ZrSi) catalysts. Experimental conditions: 0.3 g catalyst, contact time, W/F, 110 g_cat_ h (mol_GLY_)^−1^; glycerol/H_2_O/O_2_/He molar ratio of 2/40/4/54.

The effect of reaction temperature on the catalytic behaviour of the catalysts has been also studied. The most representative catalytic results are presented in Fig. 7. In general, a decrease in the selectivity to acrolein at increasing reaction temperatures is observed, together with a concomitant increase in the selectivity to carbon oxides. Nevertheless, the decrease in acrolein selectivity is lower for 15W–Si and 35W–10ZrSi catalysts, which are in fact one of the most selective materials. In addition, the selectivity to other by-products is also lower for these two catalysts (Fig. 7).

**Fig. 8:**
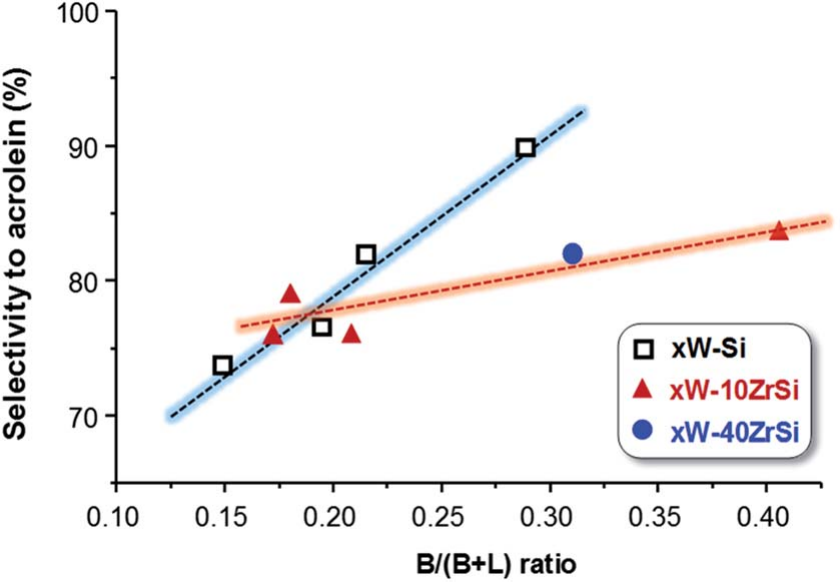
Variation of acrolein yield as a function of the proportion of surface Brønsted acid sites (B/(B + L) ratio) in the catalysts.

Interestingly, there exists a correlation between the nature of surface acid sites in the materials and the selectivity to acrolein (Fig. 8). According to this, the formation of acrolein *via* dehydration reaction is favoured as the Brønsted-type acid characteristics of the surface increase (*i.e.* at higher B/(B + L) ratio). Indeed, a higher number of Lewis acid sites could promote the re-adsorption of the product, leading to consecutive reactions to form carbon oxides, heavy by-products and other oxygenated molecules.^49^

In addition, Zr-containing catalysts follow a similar trend, although the selectivity to acrolein does not increase as drastically with Brønsted nature of the surface as in the case of *x*W–Si series (Fig. 8). As it has been pointed out by TPD-NH_3_, the addition of Zr increases the strength of surface acid sites (Fig. 3B), what it can also promote the readsorption of acrolein, leading to heavy by-products and carbon oxides, thus decreasing slightly the yield to acrolein.

**Fig. 9:**
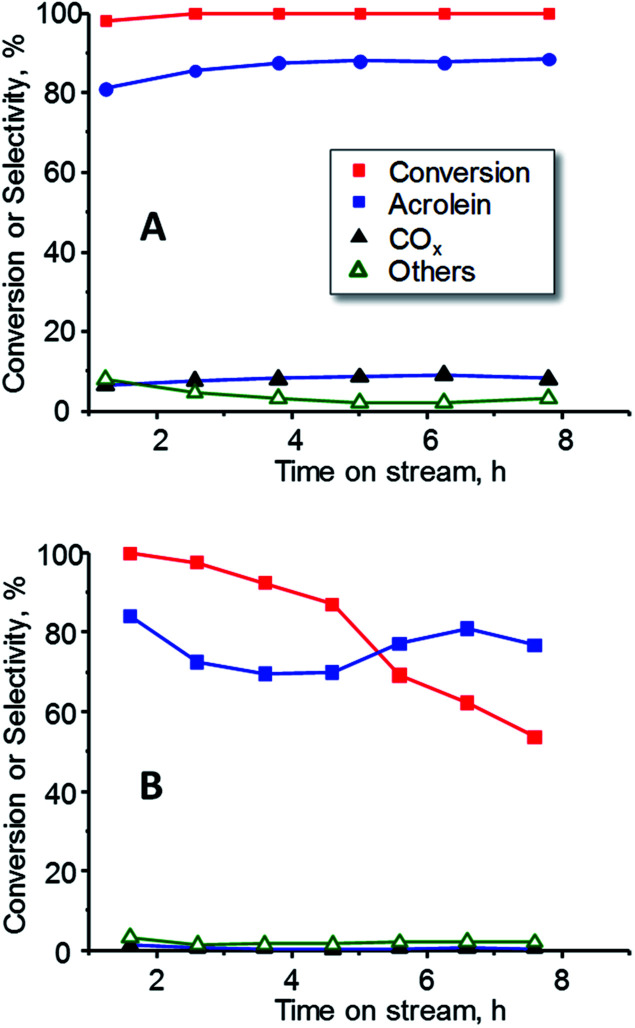
Variation of glycerol conversion and selectivity to acrolein, CO_X_ and other reaction products with time on steam during the dehydration of glycerol over 15W–Si catalysts in the presence (A) or the absence (B) of oxygen in the feed. Experimental conditions as in Fig. 7.

In order to study the effect of oxygen in the reaction, the catalytic performance of 15W–Si in glycerol dehydration was examined at a constant temperature (300 °C) during 8 h, in the presence (Fig. 9A) and in the absence (Fig. 9B) of oxygen. Important differences have been found, regarding both the glycerol conversion and the selectivity to acrolein. When oxygen is present in the feed, total conversion of glycerol and a constant selectivity to acrolein (*ca.* 85%) were observed during the 8 h of reaction (Fig. 9A). However, when oxygen is not present, the conversion of glycerol progressively decreases from 99% to 55% after 8 h of reaction, while the selectivity to acrolein varied in the range 70–85% (Fig. 9B). These results are in good agreement to previous ones in which it has been observed that co-feeding oxygen could drastically reduce catalyst deactivation and prevent the formation of acetol as by-product.^50,51^

### 3.3. General remarks

The results presented here demonstrate that it is possible to prepare active and selective mixed oxides catalysts, *i.e.* WO_3_–SiO_2_ and WO_3_–ZrO_2_–SiO_2_, for the aerobic transformation of glycerol to acrolein by one-pot non-hydrolytic sol–gel method, by optimizing both W-and Zr-contents. It is noteworthy that acrolein yields higher than 90% have been obtained with the optimal catalyst.

Catalysts with W-content between 15-35 at% of W atoms were the more selective ones, for both W–Si–O catalysts or W–Zr–Si–O catalysts with a Zr/(Zr + Si) atomic ratio of *ca.* 0.1. The maximum selectivity to acrolein observed using the optimal catalysts was *ca.* 90% at a glycerol conversion of 100%. W–Si–O and W–Zr–Si–O catalysts show acid sites, with low and medium–high acid strength in which the composition of the catalyst strongly influences the acid characteristics. More interestingly, and considering the FTIR spectra of adsorbed pyridine, we can conclude that the most selective catalysts, *i.e.* 35W–Si and 35W–10ZrSi catalysts, are those presenting the higher concentration of Brønsted acid sites. In this way, and according to the results of Fig. 8, the higher the proportion of Brønsted acid sites, the higher the selectivity to acrolein is. W-free samples present low selectivity to acrolein, since tungsten is tightly related to the formation of Brønsted acid sites and pure silica is extremely unselective towards acrolein (see Table 1). However, an excess of W has also shown a decrease in the proportion of Brønsted acid sites and consequently a drop off the selectivity to acrolein. Therefore, a trade-off in the tungsten loading is necessary to achieve the highest acrolein formation. Thus, the maximum is obtained at intermediate W-loadings.

On the other hand, the acrolein formation also decreases when the Zr-content of the catalysts increases. This is due to the lower proportion of Brønsted acid sites (lower B/(B + L) ratio) in catalysts with high zirconium content. Then, at high Zr-loadings the formation of carbon oxides and other oxygenated by-products are favoured to the detriment of the acrolein formation.

It must be noted that just the amount of acid sites does not determine neither the catalytic activity nor the acrolein formation. In fact, no correlation has been observed between the amount of ammonia adsorbed in the TPD experiments and the glycerol conversion (or the selectivity to acrolein).

According to the XPS results, octahedral W^6+^ sites are mainly present on the catalyst surface (Fig. 5 and S7†). Although this can be achieved in pure WO_3_ sample, it is clear that the presence of SiO_2_ or SiO_2_–ZrO_2_ favours the appearance of WO_3_ phases with a higher dispersion. In this way, it has been recently reported that WO_3−*x*_ crystal with hexagonal tungsten bronze (HTB) structure, are more active and selective than monoclinic WO_3_.^49^ However, an alternative way to improve catalytic performance of this type of materials could be to promote a higher dispersion of monoclinic WO_3_ by incorporating a metal oxide diluent (as SiO_2_ of Zr–Si–O mixed oxides with low Zr-content). In this way, the one-pot non-hydrolytic sol–gel method is an effective way for preparing active, selective and stable catalysts for the aerobic transformation of glycerol to acrolein.

## 4. Conclusions

In conclusion, the synthesis and characterization of W–Si–O and W–Zr–Si–O catalysts, with different W- and/or Zr-contents, prepared by non-hydrolytic sol gel method has been reported. These catalysts show high catalytic activity and selectivity to acrolein during the aerobic transformation of glycerol, with yields to acrolein between 40 and 90%, which strongly depend on the catalyst composition.

A parallelism between the selectivity to acrolein and the proportion of Brønsted acid sites in catalysts has been observed. In this way, samples with the highest B/(B + L) acid sites ratio, *i.e.* presenting W-content in the range 15–35%, and a Zr/(Zr + Si) ratio between 0 and 0.1 are the most selective ones. This is in line with results obtained with other catalytic systems, in which the proportion of Brønsted acid sites improves the catalytic performance in the dehydration of glycerol to acrolein.^52^

In addition, the yield to acrolein decreases when increasing the Zr-content in the catalysts, mainly because of their lower B/(B + L) ratio. Accordingly, the higher the Zr content, the lower the proportion of Brønsted acid sites, which gives rise to a decrease in the selectivity to acrolein, favouring the formation of carbon oxides and other oxygenated by-products.

On the other hand, strong differences in catalytic performance have been observed when the reaction is carried out in the presence or in the absence of oxygen in feed. Thus, in the absence of oxygen, an important deactivation of the catalyst occurs, probably due to coke deposition on the surface.

According to our results, we can conclude that both the total concentration and proportion of surface Brønsted acid sites are the key factors to improve the selectivity to acrolein in the aerobic transformation of glycerol.

## Conflicts of interest

There are no conflicts to declare.

## Supplementary Material

RA-008-C8RA01575A-s001
