# Supplementary material for: Gas phase dehydration of glycerol to acrolein over WO3-based catalysts prepared by non-hydrolytic sol–gel synthesis
Source: RSC Adv. 2018 Apr 10;8(24):13344–52. doi: 10.1039/c8ra01575a (PMC9079827; doi:10.1039/c8ra01575a)
Supplement: RA-008-C8RA01575A-s001 [file RA-008-C8RA01575A-s001.pdf]

Electronic Supplementary Material (ESI) for RSC Advances.
This journal is © The Royal Society of Chemistry 2018

SUPPORTING INFORMATION

Gas phase dehydration of glycerol to acrolein over WO_3-based catalysts prepared by non-hydrolytic sol-gel synthesis

L. Nadji,[a,b] A. Masso,[a] D. Delgado,[a] R. Issaadi,[b] E. Rodríguez-Aguado,[c] E. Rodríguez-Castellón,[c] J.M. López Nieto [a,*]

[a] Instituto de Tecnología Química, Universitat Politècnica de València-Consejo Superior de Investigaciones Científicas, Avenida de los Naranjos s/n, 46022 Valencia, Spain.

[b] Laboratoire des Applications Energétiques de l'Hydrogène, Faculté de Technologie, Université Saad Dahlab, Blida 1, Algeria.

[c] Department of Inorganic Chemistry, University of Malaga, Campus de Teatinos, 29071, Málaga, Spain.

Table S1. Physicochemical characteristics of W-free samples, 0W-yZrSi, prepared by NHSG.

Catalyst	Zr/(Zr+Si) atomic ratio (EDX)	Surface area,	Pore diameter,	Pore volumen,	TPD-NH_3		FTIR Pyridineadsorption
	%	m^2g^{-1}	nm	cm^3g^{-1}	μmol NH_3/g	μmol NH_3/m^2	B /(B+L)
0W-Si	0	583	11.9	1.28	107	0.18	-
0W-10ZrSi	10.1	334	4.2	1.2	859.9	2.6	0.23
0W-20ZrSi	19.6	274	3.0	0.6	-	-	0.13
0W-40ZrSi	37.9	233	2.1	0.3	-	-	0.12
0W-Zr	100	50	4.5	0.1	-	-	-

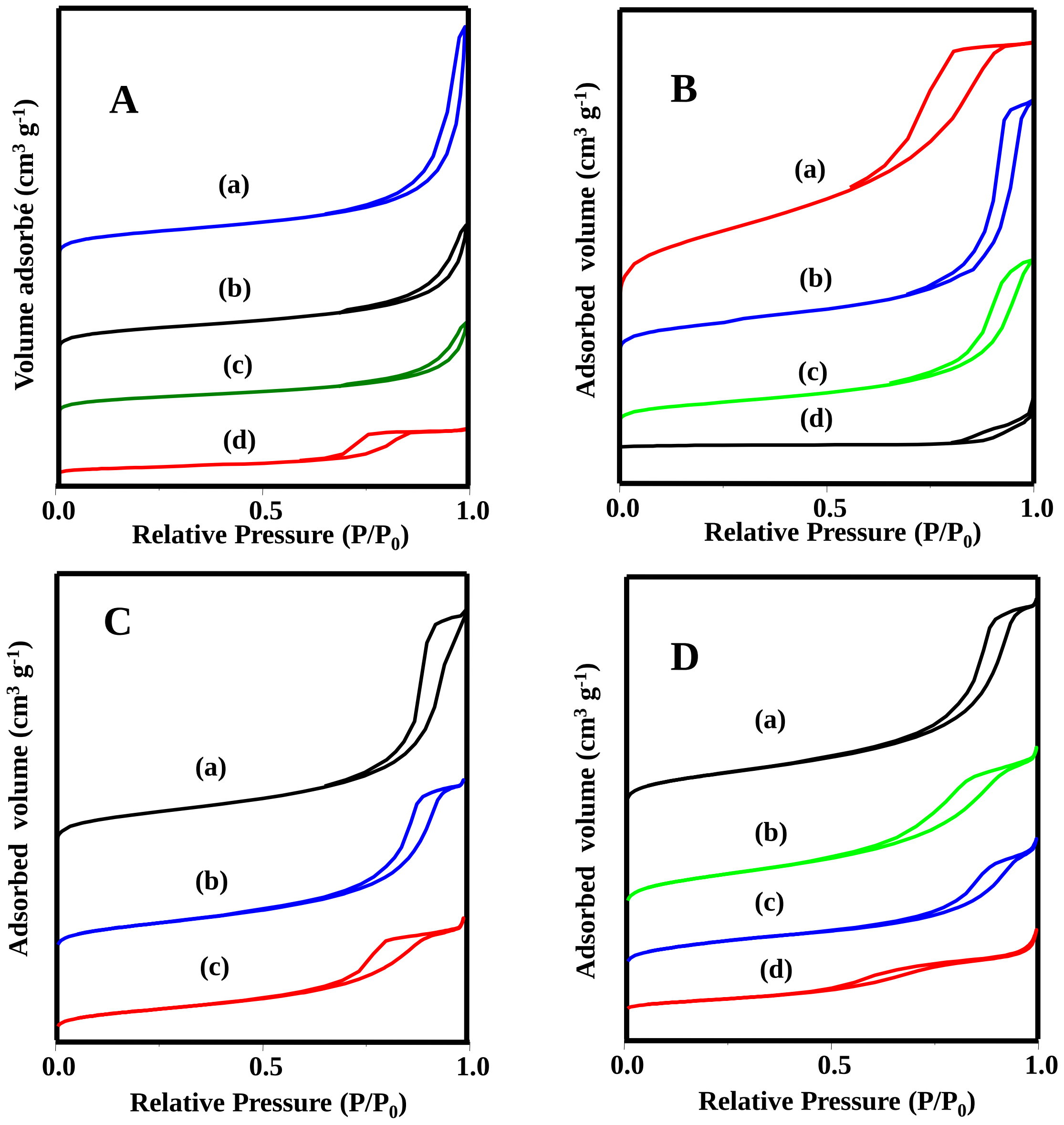

Fig.S1. Adsorption-desorption isotherm hysteresis of different catalysts prepared by non-hydrolytic sol gel method**. A)** 0W-10ZrSi (a); 0W-20ZrSi (b); 0W-40ZrSi (c) and ZrO_2 (d); **B)** 5W-Si (a); 15W-Si (b), 35W-Si (c) and WO_3 (d); **C)** 5W-10-ZrSi (a); 15W-10ZrSi (b): and 35W-10ZrSi (c); **D)** 15W10-ZrSi (a); 15W-20ZrSi] (b); 15W-40ZrSi] (c); and 15W-Zr (d).

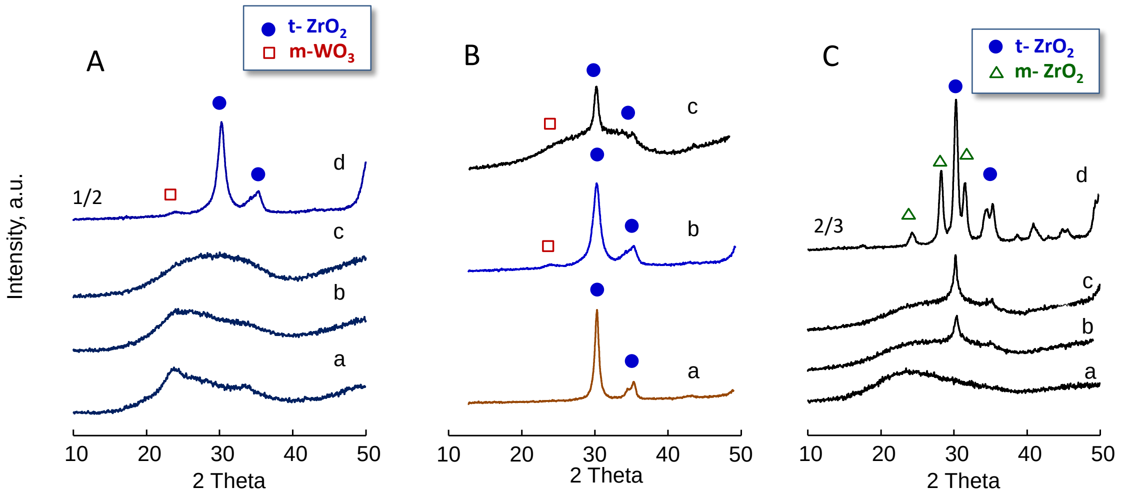

Figure S2. **A)** XRD patterns of W-containing catalysts of 15W-yZrSi series: a) 15W-10ZrSi; b) 15W-20ZrSi; 15W-40ZrSi; 15W-Zr.

B) XRD patterns of W-containing catalysts of xW-Zr series: a) 5W-Zr; b) 15W-Zr; c) 35W-Zr.

C) XRD patterns of W-free samples: a) 0W-10ZrSi; b) 0W-20ZrSi; c) 0W-40ZrSi; 0W-Zr

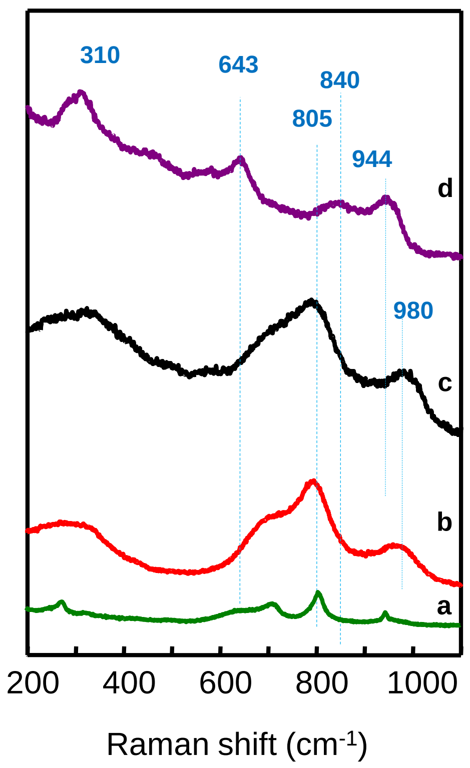

Figure S3. Raman spectra of W-containing catalysts with different Zr/Si ratio: a) 15W-Si; b) 15W-10ZrSi; c) 15W-20ZrSi; d) 15W-Zr.

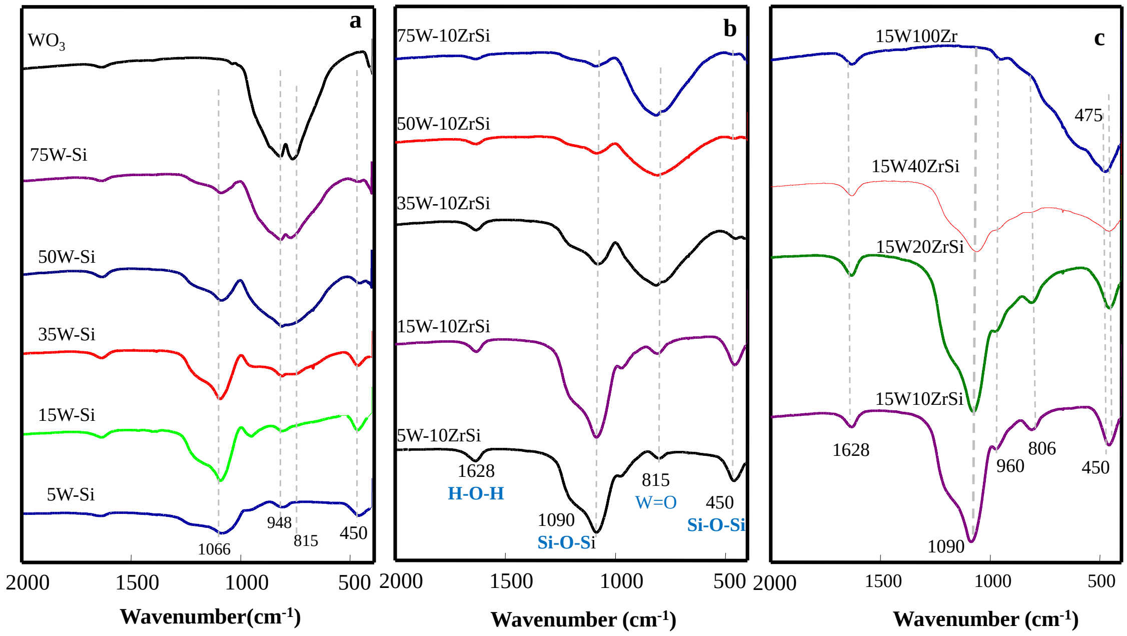

Fig. S4. FTIR Spectra of W-containing catalysts: a) xW-Si series; b) xW-10rSi series; c) xW-yZrSi series.

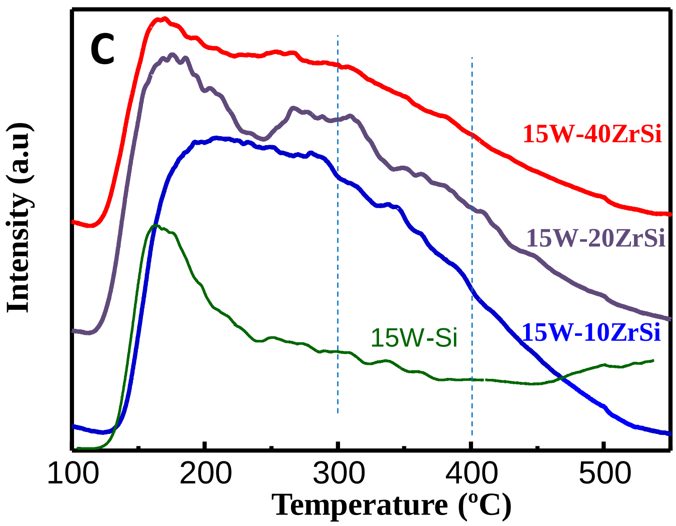

Fig. S5. TPD-NH_3 profiles of 15W-yZrSi catalysts (y = 0, 10, 20, 40).

Table S2. Acidic properties of catalysts prepared by NHSG.

Catalyst	TPD- NH_3		$B/(B+L)^a$
	$\mu mol_{NH3}\ g^{-1}$	$\mu mol_{NH3}\ m^{-2}$	FTIR (pyridine)
0W-Si	107	0.184	n.d.
5W-Si	455	0.588	0.19
15W-Si	564	1.190	0.21
35W-Si	371	1.344	0.29
50WSi	n.d.	n.d.	0.15
75W-Si	n.d.	n.d.	n.d.
WO_3	57.2	1.907	n.d.
0W10ZrSi	860	2.575	n.d.
5W-10ZrSi	825	1.299	n.d.
15W-10ZrSi	817	1.865	0.18
35W-10ZrSi	790	2.959	0.40
50W-10ZrSi	604	5.345	n.d.
75W-10ZrSi	n.d.	n.d.	n.d.
15W-20ZrSi	712	2.132	n.d.
15W-40ZrSi	590	2.554	0.31
15W-Zr	378	2.681	n.d.

[a] B: amount of Brönsted acid sites, L: amount of Lewis acid sites

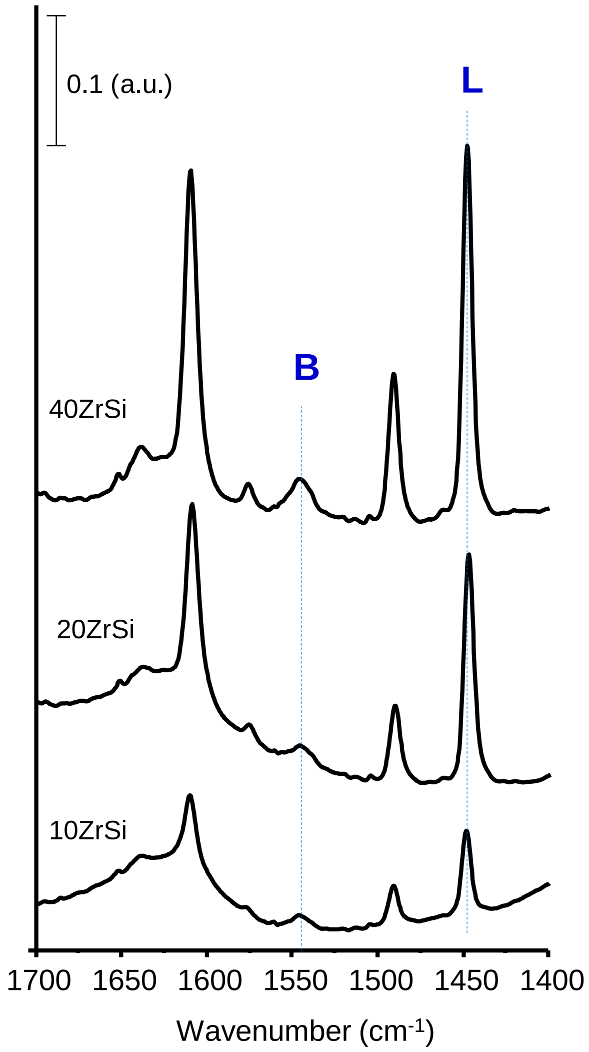

Fig. S6. FTIR of adsorbed pyridine spectrum of W-free samples, i.e. yZrSi materials.

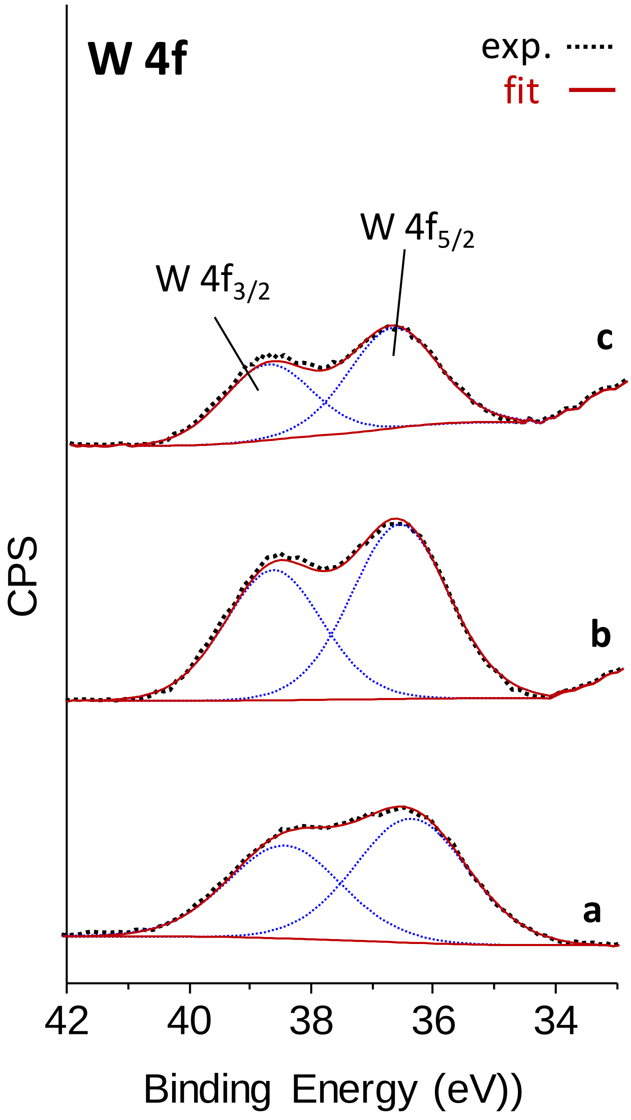

Fig. S7. W 4f core-level XPS spectra of 15W-yZrSi catalysts: a)15W-20ZrSi; b)15W40ZrSi; and c) 15W-Zr.

Table S3. Catalytic performance of WSi catalysts prepared by NHSG.

Samples	Conversion (%)	Yield Acrolein (%)	Selectivity						C-balance (%)
			Acrolein	Acetaldehyde	Acrylic Acid	Acetic Acid	COx	Others	
0W-Si	71.0	5.9	8.3	0.0	0.1	0.1	16.6	9.1	38.0
5W-Si	90.8	69.8	76.8	0.8	0.4	1.7	2.6	2.7	93.6
15W-Si	100	82.2	82.2	2.5	0.8	6.0	4.1	0.8	96.3
35W-Si	100	90.1	90.1	3.0	0.2	5.5	4.1	0.2	103.1
50W-Si	100	74.0	74.0	3.5	0.8	5.8	9.5	0.1	93.8
75W-Si	100	74.4	74.4	3.1	0.9	8.2	9.9	0.8	97.2
WO_3	99.8	78.9	79.0	2	0.1	0.7	10.4	0.6	93.8

1) Experimental conditions: 0.3 g catalyst; contact time, W/F, of **110**$g_{cat}h$ $(mol_{glycerol})^{-1}$; Glyc/H_2O/O_2/He molar ratio of 2/40/4/54; Data measured after 90 min. on stream.
2) Carbon oxides, CO and CO_2.

Table S4. Catalytic performance of xW-10ZrSi and 15W-yZrSi catalysts prepared by NHSG. [1]

Samples	W-content (%) [2]	Zr-content (%) [3]	Glycerol Conversion (%)	Yield Acrolein (%)	Selectivity (%)						C-balance (%)
					Acrolein	Acetaldehyde	Acrylic Acid	Acetic Acid	CO_x[4]	Others	
0W-10ZrSi	0	10.1	78.2	53.0	67.8	8.9	0.5	9.7	6.7	9.1	38.0
5W-10ZrSi	3.6	9.1	88.4	67.3	76.1	8.8	0.2	4.2	2.3	9.2	103
15W-10ZrSi	15.1	9.5	99.9	79.1	79.1	5.6	0.3	3.2	2.6	6.0	99.5
35W-10ZrSi	36.6	12.2	99.1	83.1	83.8	5.8	0.1	7.2	3.5	1.2	100.6
50W-10ZrSi	45.8	12.2	99.6	76.2	76.5	2.6	0.4	5.6	4.5	1.4	91.1
75W-10ZrSi	71.2	10.4	100	76.9	76.9	3.1	0.6	7.0	9.1	0.7	97.5
15W-20ZrSi	13.9	18.0	100	75.0	75.0	3.46	0.25	3.06	2.3	4.1	90.8
15W-40ZrSi	15.1	39.0	100	82.4	82.3	5.58	0.11	2.33	2.4	3.9	99.3
15W-Zr	13.3	100	100	70.2	70.2	4.60	0.39	7.97	5.1	3.1	92.8

1) Experimental conditions: 0.3 g catalyst, contact time, W/F, 110g_{cat}h ($mol_{glycerol}$)$^{-1}$; Glyc/H_2O/O_2/He molar ratio of 2/40/4/54; Data measured after 90 min. on stream; 2) W-content as W/(W+Zr+Si) atomic ratio in %; 3) Zr-content as Zr/(Zr+Si) ratio in %; 4) Carbon oxides, CO and CO_2.

Table S5. Catalytic performance of pure and mixed oxides catalysts prepared by NHSG. [1]

Samples	Glycerol Conversion (%)	Yield Acrolein (%)	Selectivity (%)						C-balance (%)
			Acrolein	Acetaldehyde	Acrylic Acid	Acetic Acid	CO_x [2]	Others	
0W-Si	71.0	5.9	8.3	0.0	0.1	0.1	16.6	9.1	38.0
0W-10ZrSi	78.2	53.0	67.8	8.9	0.5	9.7	6.7	0.9	100.4
0W-20ZrSi	99.9	48.29	48.32	2.1	0.1	2.9	1.4	11.8	67.1
0W-40ZrSi	98.1	38.3	39.0	2.7	0.2	2.8	1.6	23.7	70.1
0W-Zr	89.8	13.1	14.6	2.8	2.0	1.3	7.7	40.5	69.0

1) Data measured after 90 min. on stream. Experimental conditions: 0.3 g catalyst; contact time, W/F, of **110**$g_{cat}h\ (mol_{GLY})^{-1}$; Glycerol/$H_2O$/$O_2$/He molar ratio of 2/40/4/54.
2) Carbon oxides, CO and CO_2.
